# Predatory Bacteria Select for Sustained Prey Diversity

**DOI:** 10.3390/microorganisms9102079

**Published:** 2021-10-02

**Authors:** Ramith R. Nair, Gregory J. Velicer

**Affiliations:** 1 Department of Medical Biochemistry and Microbiology, Uppsala University, 75234 Uppsala, Sweden; 2 Institute for Integrative Biology, ETH Zürich, 8092 Zürich, Switzerland; gregory.velicer@env.ethz.ch

**Keywords:** pretator-prey coevolution, antagonism, mucoidy, predatory bacteria, bacterial predation, prey diversity, negative frequency dependence, experimental evolution, MyxoEE-6

## Abstract

Predator impacts on prey diversity are often studied among higher organisms over short periods, but microbial predator-prey systems allow examination of prey-diversity dynamics over evolutionary timescales. We previously showed that *Escherichia coli* commonly evolved minority mucoid phenotypes in response to predation by the bacterial predator *Myxococcus xanthus* by one time point of a coevolution experiment now named MyxoEE-6. Here we examine mucoid frequencies across several MyxoEE-6 timepoints to discriminate between the hypotheses that mucoids were increasing to fixation, stabilizing around equilibrium frequencies, or heading to loss toward the end of MyxoEE-6. In four focal coevolved prey populations, mucoids rose rapidly early in the experiment and then fluctuated within detectable minority frequency ranges through the end of MyxoEE-6, generating frequency dynamics suggestive of negative frequency-dependent selection. However, a competition experiment between mucoid and non-mucoid clones found a predation-specific advantage of the mucoid clone that was insensitive to frequency over the examined range, leaving the mechanism that maintains minority mucoidy unresolved. The advantage of mucoidy under predation was found to be associated with reduced population size after growth (productivity) in the absence of predators, suggesting a tradeoff between productivity and resistance to predation that we hypothesize may reverse mucoid vs non-mucoid fitness ranks within each MyxoEE-6 cycle. We also found that mucoidy was associated with diverse colony phenotypes and diverse candidate mutations primarily localized in the exopolysaccharide operon *yjbEFGH*. Collectively, our results show that selection from predatory bacteria can generate apparently stable sympatric phenotypic polymorphisms within coevolving prey populations and also allopatric diversity across populations by selecting for diverse mutations and colony phenotypes associated with mucoidy. More broadly, our results suggest that myxobacterial predation increases long-term diversity within natural microbial communities.

## 1. Introduction

Predation is one of the most common forms of inter-specific antagonism [1]. Under predation pressure, prey face the dual challenges of optimizing their own acquisition and use of resources for growth and reproduction while avoiding being killed or injured by predators. This dilemma has been shown to play an important role in the ecology and evolution of diverse prey species, including among plants [2], animals [3] and microorganisms [4].

Bacteria fall prey to a wide variety of predators, including unicellular eukaryotes [5], amoebae [6], nematodes [7], and even other bacteria [8,9]. Several studies have shown that over short time periods, microbial predators can elicit phenotypic responses providing resistance against predatory killing. These include filamentation [10], biofilm formation [11], sporulation [12] and production of various extracellular compounds [13] (reviewed in detail in [14]). Although these mechanisms can help thwart predation, many are part of a repertoire of responses that also protect against other stresses, making the degree to which they are selected specifically by predation unclear. Longer studies of predator-prey interactions make it possible to identify evolutionary-scale responses of prey specific to interaction with predators and to characterize their temporal evolutionary dynamics.

Long-term coevolution studies have examined the emergence and subsequent evolution of adaptive defensive traits of bacteria under attack by phage [15,16,17], but few have done so with predatory bacteria. Such coevolutionary studies with phage have shown that bacteria adapt to phage predation by modifying molecules involved in phage adsorption or by producing extracellular polysaccharides to restrict access to the cell surface. However, the reproductive rate of phage is generally much higher than that of its prey, such that dynamics and mechanisms of coevolution between bacteria and phage likely differ greatly from those between bacterial prey and slower-growing predatory bacteria.

Myxobacteria are soil- and sediment-dwelling bacteria that kill and consume diverse other microbes, including both Gram+ and Gram– bacteria and fungi, by mechanisms that remain poorly understood [18,19,20]. Because of this broad prey range, predation by myxobacteria is predicted to play significant roles in shaping the composition, structure and evolution of complex microbial communities [21].

In a predator-prey coevolution experiment with *Escherichia coli* as prey and the myxobacterium *Myxococcus xanthus* as predator recently named MyxoEE-6 [22], we previously showed that coevolving prey were under selection both for parallel losses of function in a prey outer-membrane protein (OmpT) and favoring the increase of genotypes that generate a mucoid-colony phenotype [23]. Mucoid colonies are characterized by increased opacity, generally lighter pigmentation and convex colony surfaces relative to non-mucoid colonies, although detailed mucoid-colony phenotypes may vary among conspecifics. Mucoidy is achieved by increased secretion of extracellular polysaccharides and can evolve in response to a number of biotic and abiotic challenges, including bacteriophages [15], antibiotics [24], macrophages [25] and menthol [26]. Mucoid colony-forming *E. coli* cells were found to arise frequently in MyxoEE-6 populations co-evolving with *M. xanthus*, but were largely absent from prey-only control populations [23]. Additionally, mucoidy was associated with reduced swarming and killing by the predator.

Our first study of MyxoeEE-6 and many earlier studies have shown that predation often strongly impacts prey diversity [23,27,28,29,30], but longer-term fates and dynamics of such predation-induced diversity are underexplored. Here we test whether mucoid lineages in MyxoEE-6 were on frequency trajectories predictive of long-term fixation of the phenotype or rather reveal the operation of evolutionary mechanisms preventing fixation, for example negatively frequency-dependent selection or clonal interference [31,32,33]. We then test for negative frequency dependence (NFD) of fitness in one clone pair and for cost of mucoidy that might promote extended maintenance of mucoid/non-mucoid polymorphisms. Finally, we characterize inter-population genetic and phenotypic diversity mediated by predation.

## 2. Materials and Methods

### 2.1. Predator-Prey Coevolution

MyxoEE-6 was conducted as described in Nair et al. (2019) [23]. Briefly, replicate populations of *M. xanthus* strain DK3470 and *E. coli* strain MG1655 (along with prey-only and predator-only controls) were paired and spread with glass beads on 8 mL prey-growth agar (1× M9 salts, 2 mM MgSO ${}_{4}$ , 0.1 mM CaCl ${}_{2}$ , 0.2% glucose, 1.5% agar) in 50 mL conical flasks. The flasks were incubated at 32 ${}^{{}^{\circ}}$ C for 84 h, after which they were harvested by adding 5 mL TPM buffer and shaking on an orbital shaker set at 300 rpm for 15 min. 1% of the surviving community was transferred to fresh minimal media and the cycle was repeated 25 times, allowing ∼166 generations of growth.

### 2.2. Mucoid Frequency Estimation and Clone Isolation

*Mucoid frequency estimation.* Evolved *E. coli* colonies that were of lighter pigmentation, more opaque and more convex relative to the ancestral colony phenotype on LB agar were identified as mucoid. To determine the frequency of mucoids in each replicate MyxoEE-6 community/population at different time points, aliquots of frozen stocks from respective lineages and time-points were thawed in 100 μ L TPM buffer, diluted and plated onto LB agar plates. Following overnight incubation at 32 ${}^{{}^{\circ}}$ C, 90% humidity, mucoid and non-mucoid colonies were distinguished and counted. Cycle 18 and cycle 25 populations were sampled over successive sampling periods, with each replicate sampling for each cycle performed independently at different times. Samples from cycles 6, 10, 14, 16, 20 and 22 of *E. coli* populations ME4, ME8, ME11 and ME12 were collected and plated together in the same sets of at least 3 temporally separated biological replicates. When zero mucoid colonies were present on the plate selected for counting from a dilution series, we estimated a hypothetical maximum mucoid frequency as [1/(colony *N* + 1)] to mimic a scenario in which the actual colony count *N* for the plate was increased by one and the additional hypothetical colony was mucoid. The four populations with the highest mucoid frequencies at cycle 18 (ME4, ME8, ME11 and ME12) were selected for examination at more time points to allow the greatest opportunity to accurately resolve frequency dynamics. The colonies scored to assess mucoid frequencies were not subsequently cultured or stored frozen.

*Isolation of cycle 18 mucoid and non-mucoid clones.* One mucoid and one non-mucoid clone each were isolated from cycle-18 co-evolved populations in which mucoids were detected and subsequently stored frozen. The clone pair from ME4 was used for the direct competition experiment measuring mucoid vs non-mucoid relative fitness and all nine clone pairs were used in the survival-under-predation and productivity assays and for colony-phenotype imaging. Single colonies of each phenotype were picked and streaked onto fresh LB agar plates to isolate a sub-colony and thereby purge any genetic variation that may have been present in the colony from the original plating. Isolated colonies of each phenotype were picked, grown to high density in LB liquid and samples of the resulting cultures were stored as frozen stocks (20% glycerol) for further experiments. The same mucoid and non-mucoid clone from each co-evolved population were used in the productivity and predation assays described below and were used for imaging colony phenotypes. For colony-phenotype imaging, aliquots of frozen stocks from mucoid and non-mucoid clones from nine different lineages were thawed in 100 μ L TPM buffer and 10 μ L of the suspension was spotted on an LB agar plate. The resulting colony was imaged after overnight growth at 32 ${}^{{}^{\circ}}$ C using Olympus SZX16 stereomicroscope at 0.8X magnification. The same mucoid and non-mucoid clones from cycle 18 population ME4 were also used in the relative fitness assay.

### 2.3. Productivity and Predation Assays

Experiments measuring *E. coli* mucoid vs. non-mucoid relative fitness in the presence and absence of *M. xanthus*, survival in the presence of *M. xanthus* and population size after 25 h of incubation (productivity) were performed under the same abiotic conditions as MyxoEE-6 [23]. *E. coli* cells of each type were grown overnight in LB liquid at 32 ${}^{{}^{\circ}}$ C, 300 rpm, then diluted to OD ${}_{600}$ 1.0 with LB and subsequently diluted 1:100 with TPM liquid buffer immediately prior to initiating the relevant assay. Initial and final population sizes were determined by dilution plating onto LB agar and counting colonies after overnight incubation at 32 ${}^{{}^{\circ}}$ C, 90% humidity. *M. xanthus* was grown in 8 mL CTT liquid (10 g/L casitone, 10 mM Tris pH 8.0, 8 mM MgSO ${}_{4}$ , 1 mM KPO ${}_{4}$ ) in 50 mL flasks at 32 ${}^{{}^{\circ}}$ C, 300 rpm until mid-exponential phase, when cultures were centrifuged (5000 rpm, 15 min) and resuspended in TPM buffer to a density of ∼10^9^ cells/mL. 50 μ L of the diluted prey suspension were then either mixed with 50 μ L of *M. xanthus* cell suspension at ∼10^9^ cells/mL or 50 μ L TPM buffer (productivity assays and competitions without predator) before being spread onto MyxoEE-6 prey-growth agar [23]. For the relative-fitness competition experiment, one mucoid and one non-mucoid clone each isolated at the end of MyxoEE-6 cycle 18 from the coevolution treatment ME4 were used. In those experiments, cultures of the paired *E. coli* competitors were mixed at the specified ratios after the adjustment to OD ${}_{600}$ 1.0 prior to proceeding as described above. Cultures were harvested as during MyxoEE-6. The selection rate constant was calculated as in [34] using the following formula:

(1) $$S_{ij}=\frac{1}{t}\left[ln\left(\frac{N_{i}\left(t\right)}{N_{i}\left(0\right)}\right)-ln\left(\frac{N_{j}\left(t\right)}{N_{j}\left(0\right)}\right)\right],$$

where *N ${}_{i}$ (0)* and *N ${}_{i}$ (t)* are initial and final population densities of the mucoid clone, while *N ${}_{j}$ (0)* and *N ${}_{j}$ (t)* are initial and final population densities of the competing non-mucoid clone respectively.

### 2.4. Genomic Data

Candidate mutations for causation of mucoidy were determined by comparing whole-genome sequences from two non-mucoid clones and one mucoid clone from four co-evolved populations after cycle 25 (ME4, ME8, ME11, ME12). The genomic data for these 12 clones have already been published [23] and made available elsewhere (deposited in the SRA database under BioProject accession PRJNA551936 (BioSample accessions SAMN12169214—SAMN12169315)).

### 2.5. Statistical Analysis

Frequency dependence of the selection rate constant for competition experiments between mucoid and non-mucoid clones from population ME4 was tested using one-way ANOVA with starting frequency (0.1, 0.25, 0.5) as a factor. We tested for differences in average productivity as well as killing resistance between mucoid vs. non-mucoid clones using Welch’s two-sided, two-sample *t*-tests. We tested for differences in productivity and killing resistance among mucoid clones from different coevolving populations with one-way ANOVA with population ID as a factor in both cases. Statistical analyses were performed in R [35] using R studio (version 1.3.1093) and plotted with the package ggplot2 version 3.3.4 [36].

## 3. Results

### 3.1. Mucoid Phenotypes Rise to and Remain within Intermediate Frequency Ranges

In the first study of MyxoEE-6, we reported parallel emergence of mucoid colony-forming variants in *E. coli* prey among ten out of twelve replicate populations that had co-evolved with *M. xanthus* for 18 cycles [23], whereas mucoids were largely absent from control *E. coli* populations that evolved without predators. This pattern strongly suggested that most or all mucoid genotypes detected among the co-evolved populations rose to high frequency due to selection rather than neutral drift. Indeed, mucoidy was found to be associated with reduced susceptibility to predation. However, mucoid colonies were present only as a minority after cycle 18, leaving open the question of whether, toward the end of MyxoEE-6, mucoids were (i) sweeping to fixation, (ii) decreasing toward loss due to clonal interference from non-mucoid adaptive mutants, or (iii) being maintained long-term by balancing selection while fluctuating around intermediate equilibrium frequencies.

In this study, we first compared mucoid frequencies in all MyxoEE-6 *E. coli* populations, including the 12 replicate populations that co-evolved with *M. xanthus* (ME1–ME12) and the six control replicate populations that evolved in the absence of *M. xanthus* (E1–E6), between cycles 18 and the end of the MyxoEE-6 experiment at cycle 25. Although mucoids appear to have generally decreased in frequency in many co-evolved prey populations from cycle 18 to 25, they were nonetheless present above our levels of detection in nine of the twelve co-evolved populations at cycle 25 (Figure S1). Between cycles 18 and 25, mucoids dropped below the limit of detection in two populations (ME7 and ME9) and newly rose to a detectable level in one population in which no mucoids had been found at cycle 18 (ME2, Figure S1). Thus, in total across the two time points, mucoid colony variants were observed in eleven out of the twelve coevolving prey populations and were at frequencies above the limit of detection at both time points in most. In contrast, among the six prey-only control populations, mucoidy was detected in only one replicate sample of one population at one time point (Figure S1).

**Figure 1:**
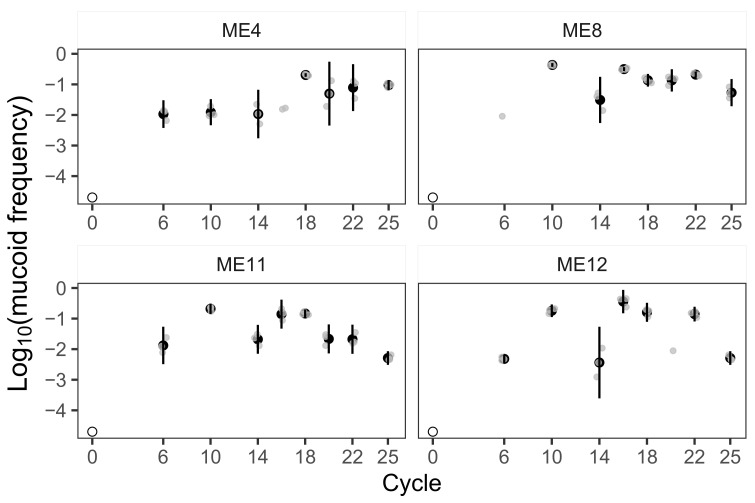
Mucoid prey rose to and persisted within intermediate frequency ranges in MyxoEE-6 in four focal populations. Frequencies of mucoid variants among the four focal coevolving prey populations estimated hypothetically at the start of MyxoEE-6 (cycle 0, open circles) and estimated directly by sampling at the end of eight MyxoEE-6 cycles (see Methods). A mucoid frequency of ∼10^−5^ at cycle 0 is shown for a hypothetical scenario in which there was one mucoid cell among the ∼10^5^ starting prey cells for each *E. coli* population. Grey dots show individual replicate estimates, black dots indicate cross-replicate means and error bars show 95% confidence intervals (*t*-distribution, three temporally separated biological replicates).

The cycle 25 results did not strongly suggest trajectories toward either fixation or loss of mucoids. We thus addressed the above hypotheses more rigorously for the four prey populations with the highest mucoid frequencies at cycle 18 by examining their mucoid frequencies at six additional MyxoEE-6 timepoints (Figure 1). In all four populations, mucoids increased several orders of magnitude from zero to detectable frequencies already by the end of cycle 6, indicating strong selection favoring mucoidy. After this commonality of rapid early increase, mucoids in the four populations fluctuated within an intermediate range of detectable minority frequencies (Figure 1).

After cycle 6, however, each of the four focal populations showed a qualitatively unique pattern of subsequent mucoid-frequency dynamics. Mucoids in the ME4 community exhibited the most stable dynamics, remaining at frequencies near 0.01 from cycles 6–16, rising ∼10-fold by cycle 18 and remaining at frequencies near 0.1 for the duration of MyxoEE-6. Mucoids in ME8, ME11 and ME12 showed strikingly similar dynamics through cycle 16, all increasing to frequencies above 0.1 by cycle 10, subsequently decreasing by cycle 14 and increasing in parallel again by cycle 16 before diverging more in their detailed dynamics through the remaining cycles.

It is noteworthy that on the one hand, none of the four populations showed any indication of mucoids approaching fixation and, on the other hand, after cycle 25, mucoids in all populations remained within or very near the respective mucoid frequency range covered from cycles 6–22. Thus, despite late decreases in three populations (ME8, ME11 and ME12), the observed patterns are consistent with, and in our view suggestive of, extended maintenance of minority mucoids by NFD of mucoid fitness.

### 3.2. A Single-Cycle Competition Experiment Reveals a Predation-Specific Advantage to Mucoidy but Not NFD

NFD of fitness might be generated by direct interactions between mucoid and non-mucoid cells within a given MyxoEE-6 growth cycle. For example, costly production of exopolysaccharides by mucoids might confer some degree of social protection from predation and thus a fitness advantage to non-mucoids, with the degree of benefit conferred correlating with mucoid frequency. In this cheating scenario, NFD-mediated maintenance of the mucoid/non-mucoid polymorphism should be manifested by a reversal of fitness ranks (e.g., [37,38]) between mucoids and non-mucoids simply as a function of frequency within a single MyxoEE-6 growth cycle.

In our first study of MyxoEE-6, we reported that fewer cells of a mucoid clone isolated from the coevolving ME4 prey population at cycle 18 are killed by the ancestral predator compared to a contemporary non-mucoid isolate [23], but direct competition experiments between these prey isolates were not performed. Here we directly competed these same two isolates in the presence and absence of predators to both confirm a predicted direct relative fitness advantage of the mucoid isolate specific to predation pressure and to test whether this fitness advantage is frequency dependent within a single competition cycle due to social cheating.

**Figure 2:**
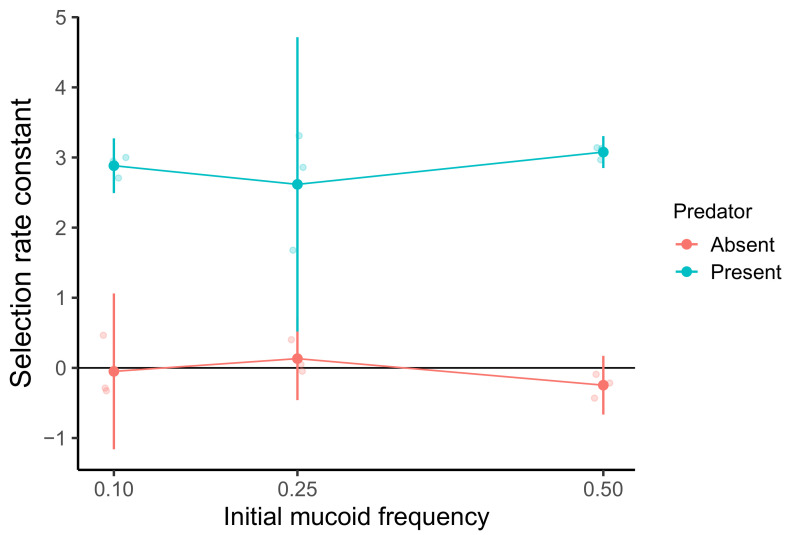
A mucoid clone outcompetes a non-mucoid clone only under predation pressure but does not exhibit negative frequency dependence. Estimates of the fitness of a mucoid clone isolated from ME4 at cycle 18 in competition relative to a contemporary non-mucoid clone (as represented by the selection-rate constant) across three initial frequencies are shown. x-axis values depict the estimated starting frequencies for the mucoid clone at the beginning of the experiment. Positive and negative values indicate estimates that the mucoid clone has higher or lower fitness than the non-mucoid clone, respectively. Smaller and larger dots represent individual-replicate and mean values, respectively. Error bars show 95% confidence intervals from three biological replicates (*t*-distribution).

The mucoid and non-mucoid prey clones were mixed at initial starting mucoid frequencies of 0.1, 0.25 and 0.5 and allowed to compete while growing under the same the experimental conditions as MyxoEE-6, except that the predator (a clone of *M. xanthus* also isolated from the ME4 community after cycle 18) was either absent or added at a population size of ∼5 × 10 ${}^{7}$ predator cells to impose predation pressure. In these assays, the mixed *E. coli* populations generally increased more than 100-fold during the competition period. As expected, the mucoid genotype had higher fitness than the non-mucoid clone in the presence of *M. xanthus* but not in its absence (Figure 2, one-way ANOVA, F ${}_{1,16}$ = 235.1, *p* = 5.49 × 10 ${}^{-11}$ ). However, NFD of fitness within a single competition cycle was not detected for this particular pair of competitors (one-way ANOVA, F ${}_{2,6}$ = 0.644, *p* = 0.558). These results thus do not support the hypothesis that mucoids fail to reach fixation due to cheating-mediated NFD. However, we note that this outcome does not generally exclude the NFD hypothesis for other experimental conditions or for other mucoid and non-mucoid competitors.

### 3.3. Mucoidy Is Associated with Low Prey Productivity in the Absence of Predators

Adaptations of prey that decrease susceptibility to predation often come at a cost to other components of fitness. We previously found no cost of mucoidy to growth rate from 5–20 h after inoculation into the MyxoEE-6 selective regime for a pair of ME4 cycle 18 clones in the absence of predators [23]. However, final population-size productivity as well as rate of population increase can impact overall fitness and we hypothesized that mucoidy might come at cost to productivity. To explore this possibility, we examined one mucoid and one non-mucoid clone each from nine of the cycle 18 coevolving populations in which mucoids were detected (ME3, ME4, ME5, ME6, ME7, ME8, ME9, ME11, ME12).

**Figure 3:**
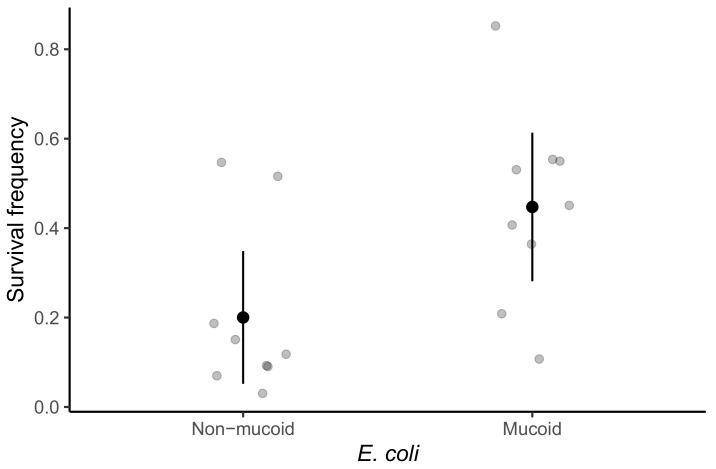
Mucoidy is associated with higher survival of encounters with *M. xanthus*. Frequencies of prey surviving encounters with *M. xanthus* are shown. Grey dots are mean survival frequency for clones from each of nine coevolving populations from which the clones were isolated at the end of cycle 18. Three independent estimates were generated for each population (Figure 5b). Black dots are means across the nine populations for each prey type and error bars show 95% confidence intervals (*t*-distribution).

**Figure 4:**
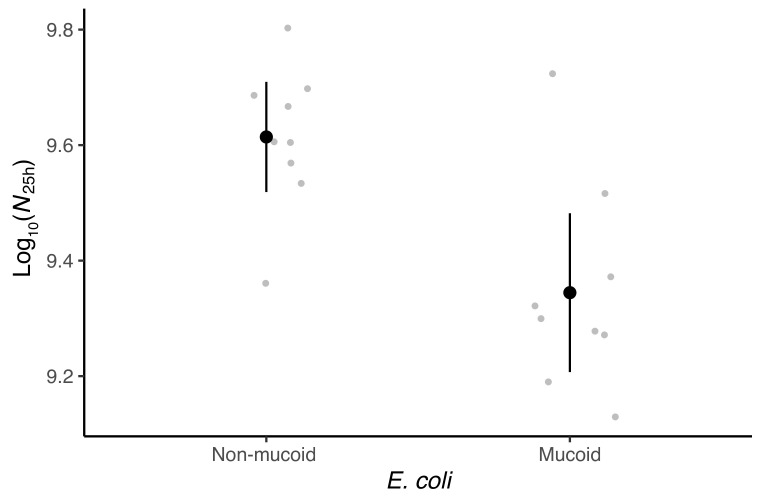
Mucoids have lower population productivity than non-mucoids in the absence of predators. Log-transformed (productivity) is shown. Grey dots are mean values from nine coevolving populations from which the clones were isolated at the end of cycle 18. Three independent estimates were generated for each population (Figure 5c). Black dots are means across the nine populations for each prey type and error bars show 95% confidence intervals from nine replicate populations (*t*-distribution).

We first confirmed that the mucoid clones are on average less susceptible to killing by *M. xanthus* than the corresponding non-mucoid clones from the same population (Figure 3, Welch’s two-sample *t*-test: *t* ${}_{15.8}$ = 2.558, *p* = 0.011), as was expected from previous results with the clone pair from population ME4 [23]. To examine the productivity-cost hypothesis, the clones were grown under MyxoEE-6 abiotic conditions for 25 h in the absence of predators and their final population sizes (productivity) determined. The average productivity of mucoid clones was found to be ∼27% lower than that of non-mucoid clones (Figure 4, Welch’s two-sample *t*-test: *t* ${}_{14.18}$ = −3.5636, *p* = 0.0015). Thus, mucoidy protects against predation, but at a cost to total productivity in the absence of predation. This productivity cost of mucoidy led us to speculate that it may mediate a form of NFD in which mucoid frequency impacts total *E. coli* productivity, which, along with and decreased susceptibility to predation by mucoids, impacts predator productivity at the end of a growth cycle, which in turn impacts mucoid vs non-mucoid relative fitness in the subsequent growth cycle. We elaborate this hypothesis further in the Discussion.

### 3.4. Mucoidy Is Phenotypically Variable

**Figure 5:**
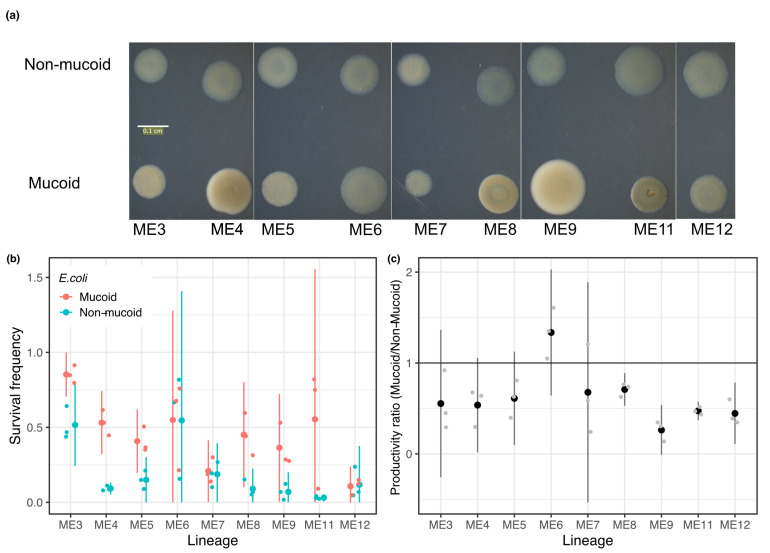
Allopatric variation among mucoid prey. (**a**) Observed mucoid (bottom row) and non-mucoid (top row) colony phenotypes on LB agar for the respective clones isolated from nine coevolving populations at the end of cycle 18 and used in the experiments reported in Figure 3, Figure 4 and Figure 5b,c. Phenotypic differences between the mucoid isolates and between mucoid vs. non-mucoid clones from the same population are yet more pronounced upon direct visual and microscopic observation than in the two-dimensional images shown here. (**b**) Frequencies of prey surviving encounters with *M. xanthus* are shown. (**c**) The relative productivity (population size after 25 hr) of single mucoid vs non-mucoid clones isolated from nine populations at the end of cycle 18 is shown. A value of 1 (solid black line) indicates equal productivity. (**b**,**c**) Small dots are values from individual replicates and large dots are inter-replicate means. Errors bars show 95% confidence intervals (*t*-distribution, n = 3).

We observed visually that colonies of the nine mucoid clones isolated from different lineages at the end of cycle 18 were phenotypically diverse, varying with regard to colony size, opacity, color and degree of phenotypic differentiation from non-mucoid colonies from their respective populations (Figure 5). We therefore tested for quantitative variation among these clones with respect to susceptibility to predation and productivity. While the mucoid clones are collectively less susceptible to killing by *M. xanthus* than non-mucoids (Figure 3), the degree of resistance to killing varied significantly among the nine mucoid clones tested here (Figure 5b, one-way ANOVA, F ${}_{8,18}$ = 3.988, *p* = 0.007). Additionally, while mucoids collectively have lower productivity than non-mucoids in the absence of predation (Figure 4), the isolated mucoid clones varied significantly in their productivity relative to contemporaneous non-mucoid clones isolated from the same prey population (Figure 5c), one-way ANOVA, F ${}_{8,18}$ = 4.4158, *p* = 0.004). Thus, while mucoidy in general is clearly selected by bacterial predation, the broader categorical phenotype can be associated with a diversity of detailed colony phenotypes and predation-related parameter values.

### 3.5. Candidate Mutations for Mucoidy

The observed variation in mucoid phenotypes suggested that distinct prey lineages may have followed different genomic routes to mucoidy, with distinct causal mutations having different phenotypic effects. Alternatively, the variable mucoid phenotypes might have resulted from differences in epistatic interactions between shared mutations or mutation targets (genes or gene pathways) and other evolved mutations in the same genetic background. To generate hypotheses regarding genetic causation of mucoidy in MyxoEE-6, we compared whole-genome sequences of one mucoid and two non-mucoid clones each from the four focal coevolved prey populations examined here for mucoidy dynamics across MyxoEE-6 (ME4, ME8, ME11, ME12) [23]. Mucoid clones from all four populations each had a mutation in one gene in the *yjbEFGH* operon, with only one gene (*yjbH*) mutated in more than one population (Table 1). In contrast, no mutations in this operon were found in any of the non-mucoid colonies, strongly suggesting that mutations in this operon confer mucoidy. This operon encodes proteins involved in the production of an uncharacterized extracellular polysaccharide [39]. Additionally, the mucoid clone from ME4 also has a mutation upstream of the gene *rcsA* (Table 1). Because *rcsA* is a transcriptional regulator of colanic acid capsular biosynthesis [40], this mutation may contribute to mucoidy in this clone.

## 4. Discussion

By imposing predatory selection on prey, predators can increase diversity within prey communities and populations by diverse mechanisms. For example, keystone predators can promote diversity by targeting the most dominant prey species and thereby increasing resources available for other prey species [27,28,29,41,42]. Predation can ameliorate competitive exclusion among competing prey species by promoting prey that are less competitive at low predation pressure but less susceptible to predation by a dominant predator [10,43]. This can result in a negative feedback loop in which predation drives increase of less-susceptible prey, eventually resulting in reduced predator population size (and thus reduced predation pressure), which in turn reverses prey fitness ranks back in favor of prey that are more competitive at low predation pressure [28]. Predators can also maintain polymorphisms among prey through apostatic selection [30,44,45] and across environmental gradients by selecting for different prey alleles in different environmental contexts [46]. While such effects of predation on prey diversity have been investigated in a broad array of organisms, predatory bacteria are understudied in this regard.

Having previously shown that predatory bacteria select for mucoid variants of prey during coevolution [23], here we tested whether mucoid frequency dynamics indicate or suggest longer-term fixation, loss or intermediate persistence of mucoidy, especially in four focal populations of MyxoEE-6. Mucoid frequencies increased several orders of magnitude early in the experiment until reaching detectable frequencies already by cycle 6 (Figure 1). At this rate of increase, mucoids should have easily reached fixation during MyxoEE-6 in the absence of clonal interference or negative frequency dependence, but did not. Among the four focal populations, mucoids ceased increasing after reaching detectable frequencies and never reached majority status at sampled time points. After reaching their maximum frequencies, rather than decreasing continuously to undetectable frequencies, as might be expected under clonal interference by non-mucoid adaptive genotypes, mucoids fluctuated within detectable frequencies ranges to the end of MyxoEE-6, an outcome suggestive of negatively frequency-dependent selection.

In light of the above results, we designed an experiment to test for such NFD between a mucoid and a non-mucoid clone from population ME4 (cycle 18) resulting from immediate effects of prey-type frequency on prey-type fitness that would be observable over the course of a single cycle of MyxoEE-6 community growth. Such single-cycle NFD could result, for example, if non-mucoids can cheat on mucoids with respect to EPS production [47], not incurring the cost of excess EPS production borne by mucoids but receiving some benefit of such production by neighboring mucoid cells with respect to predation susceptibility. However, this experiment did not detect such NFD between these two focal prey clones, despite confirming a general fitness advantage to the mucoid clone specific to the presence of predation pressure (Figure 2). This outcome might suggest that mucoid vs. non-mucoid fitness is in fact not generally dependent on frequency in a predation-dependent manner, but it is premature to exclude the NFD hypothesis absent further experiments. It remains possible that the observed fitness relationships between the particular ME4 clones chosen for this experiment are not representative of those between most mucoid and non-mucoid genotypes and/or that details of the experimental conditions under which the competition experiment was performed (e.g., starting predator density) prevent the manifestation of NFD that did actually occur during MyxoEE-6. Alternatively, the productivity cost of mucoidy demonstrated here (Figure 4 and Figure 5c) suggested to us the possibility of a distinct form of NFD that, if it occurs, would only play out over multiple growth cycles.

We speculate that the productivity cost and predation-resistance of mucoidy have the potential to collectively mediate NFD of mucoid fitness by reducing predator numbers when mucoids are sufficiently frequent in one growth cycle, which may in turn impact mucoid vs. non-mucoid fitness in the subsequent growth cycle. Under MyxoEE-6 conditions, *E. coli* grows much faster than *M. xanthus* and *M. xanthus* is completely dependent on *E. coli* for growth. For these reasons, we hypothesize that *E. coli* populations generally reached carrying capacity within each growth cycle before *M. xanthus* predator populations increased sufficiently to impose strong predation pressure that would selectively favor mucoids. Due to the productivity cost of mucoidy, non-mucoids may have generally outcompeted mucoids upon completion of prey growth earlier in each cycle, while mucoids may have often outcompeted non-mucoids later in each cycle, after predator density had increased sufficiently to impose strong predation pressure and thus favor mucoidy. In this scenario, sufficiently high late-cycle predation pressure promotes the net increase of mucoids over the entire cycle, despite a disadvantage of mucoids upon completion of prey growth before predators increase to large population sizes. However, mucoid frequency in one MyxoEE-6 cycle might impact the degree of late-cycle predation pressure in the next cycle if predators exhibit lower productivity on mucoid cells than non-mucoids.

This latter scenario would require first that predators exhibit lower productivity on mucoids than on non-mucoids, a plausible scenario given that mucoid prey have lower productivity and are less susceptible to predatory death. It would also require that lower predator productivity caused by relatively high mucoid frequency in one cycle decreases total predation pressure over the next cycle sufficiently to give non-mucoids a net whole-cycle advantage over mucoids in that subsequent cycle. This hypothetical scenario involves a negative feedback loop analogous to such loops proposed or demonstrated to maintain diversity in other predator-prey systems [28]. A test of this hypothesis with respect to MyxoEE-6 would require additional experiments to examine whether these requirements are often met across a range of mucoid vs non-mucoid genotype combinations.

Mucoid phenotypes in *E. coli* are associated with increased production of exopolysaccharides, especially colanic acid (CA-EPS) [48,49] and have been linked with mutations in *rcs*, *yjb* and *yrf* genes [40,48]. Given that all sequenced mucoid *E. coli* clones carried mutations in the *yjbEFGH* operon, while none of the non-mucoid clones had such a mutation, it appears that mucoidy in MyxoEE-6 was achieved predominantly by mutation of this operon, which is involved in the synthesis of an uncharacterised exopolysaccharide [39]. Interestingly, deletion of this operon in a Δ *rpoS* background has been shown to result in CA-EPS synthesis and mucoidy [48]. Apart from the mutations in *yjbEFGH*, the mucoid strain from lineage ME4 had an additional mutation upstream of *rcsA*, which is a known positive regulator for colanic acid synthesis [40,50]. Thus, mutations in *yjbEFGH* and *rcsA* are likely to mediate the apparent tradeoff between resistance to *M. xanthus* predation and productivity, although additional genetic experiments would be required to demonstrate this directly. Collectively, mucoidy seems to have been achieved largely through mutating the same operon, but in different genes and sites. These distinct mutations may have themselves generated the phenotypic variation among mucoid genotypes documented here, or they may have interacted with other mutations to generate such diversity.

Natural microbial communities contain myriad species of prey and predators co-existing in complex food chains and webs [51,52,53]. The shifting balance of selection imposed by predation vs resource utilisation is likely to contribute to this diversity [28,41,43]. Our results with simple synthetic bacterial communities initially derived from just one prey genotype and one predator genotype suggest that both sympatric and allopatric prey diversity that is evolutionarily induced by bacterial predators can be long-lived. This suggests more strongly than previous studies that predators of microbes and myxobacteria in particular promote greater intra-specific diversity across many prey types over long evolutionary time scales than would co-exist in the absence of predation. Future experiments with more complex communities are required to test this prediction.

At the inter-specific level, it is now well understood that variable levels and forms of intraspecific diversity impact community evolution (e.g., [54,55,56]). Thus, myxobacteria are likely to shape microbial community composition and structure both directly through differential predation of distinct species [21] and indirectly by shaping long-term patterns of intraspecific diversity that in turn influence inter-specific fitness relationships.

## Figures and Tables

**Table 1:** Mutation candidates for causation of mucoidy in cycle 25 mucoid isolates from the four focal co-evolved populations.

Gene	Mutation	Gene Function	Population
*rcsA*	deletion (1 bp)	transcriptional regulator of colanic acid capsular biosynthesis	ME4
*yjbG*	SNP	extracellular polysaccharide export outer-membrane associated protein	ME4
*yjbE*	insertion (+9 bp)	extracellular polysaccharide production threonine-rich protein	ME8
*yjbH*	SNP	DUF940 family extracellular polysaccharide protein	ME11
*yjbH*	insertion (+8 bp)	DUF940 family extracellular polysaccharide protein	ME12

## Data Availability

Raw data presented in figures available at Figshare (doi:10.6084/m9.figshare.16726465).

## Supplementary Materials

The following are available online at https://www.mdpi.com/2076-2607/9/10/2079/s1, Figure S1: Mucoid-frequency estimates for all evolved MyxoEE-6 *E. coli* populations after cycles 18 and 25.
