# Supplementary material for: Predatory Bacteria Select for Sustained Prey Diversity
Source: Microorganisms. 2021 Oct 2;9(10):2079. doi: 10.3390/microorganisms9102079 (PMC8540638; doi:10.3390/microorganisms9102079)
Supplement: Supplementary file 1 [file microorganisms-09-02079-s001.zip › microorganisms-1324015-supplementary.pdf]

Supplementary information:

Predatory bacteria select for sustained prey diversity

Ramith R. Nair and Gregory J. Velicer

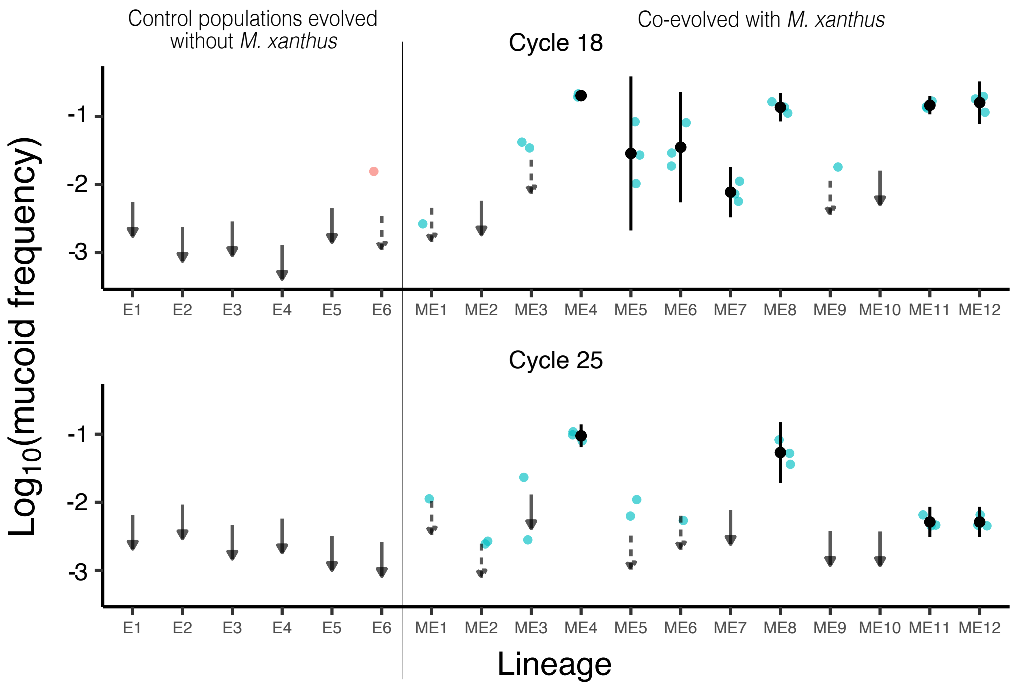

Figure S1: **Mucoid-frequency estimates for all evolved MyxoEE-6 E. coli populations after cycles 18 and 25.** Colored dots represent individual replicate estimates and black dots represent mean estimates for populations that yielded mucoid colonies in all three replicate platings. Error bars show 95% confidence intervals (t-distribution). Dilution platings for which zero mucoid colonies were present are represented by downward pointing arrows. Solid arrows indicate cases for which all three replicate plates had zero mucoids. In these cases, the top end of the arrow indicates the maximum possible mean mucoid frequency under an imaginary scenario in which one additional colony had been counted for each plate and each of those additional colonies was mucoid. Dashed arrows indicate populations for which only one or two replicates had zero-counts, in which case the top end of the arrow indicates the same as for the solid arrows, except either for one replicate or the average of two replicates.
